# Movement deficits and neuronal loss in basal ganglia in TRPC1 deficient mice

**DOI:** 10.18632/oncotarget.12567

**Published:** 2016-10-11

**Authors:** Kaiwu He, Fei Qi, Chunni Guo, Shuqin Zhan, Hua Xu, Jianjun Liu, Xifei Yang

**Affiliations:** ^1^ College of Pharmacy, Jinan University, Guangzhou, China; ^2^ Key Laboratory of Modern Toxicology of Shenzhen, Shenzhen Center for Disease Control and Prevention, Shenzhen, China; ^3^ Department of Respiratory Medicine, Chinese People's Liberation Army General Hospital, Beijing, China; ^4^ Department of Neurology, Shanghai First People's Hospital Affiliated to Shanghai Jiaotong University, Shanghai, China; ^5^ Department of Neurology, Xuanwu Hospital of Capital Medical University, Beijing, China

**Keywords:** TRPC1, basal ganglia, movement disorder, neuronal loss, Pathology Section

## Abstract

Transient receptor potential cation (TRPC) channel proteins are abundantly expressed in brain. However, the functions of these TRPC proteins such as TRPC1 are largely unclear. In this study, we reported that TRPC1 deficiency caused movement disorder as measured by swimming test, modified open field test and sunflower seeds eating test. Immunofluorescent staining showed significant loss of both NeuN-positive cells and tyrosine hydroxylase (TH) -positive cells in the caudate putamen (CPu), the external globus pallidus (GPe), and the substantia nigra pars reticulata (SNr) in 5-month-old TRPC1 knockout mice (TRPC1^−/−^) compared to the wild type (WT) mice. TUNEL staining further revealed that TUNEL-positive cells were significantly increased in the CPu, GPe, and SNr of TRPC1^−/−^ mice. Taken together, these data suggests that TRPC1 is involved in the control of motor function by inhibiting the apoptosis of neuronal cells of basal ganglia.

## INTRODUCTION

The basal ganglia are a group of subpalial nuclei which have an important part in motor, emotional, and cognitive functions. Though the term “basal ganglia” has been defined in many different ways, we choose to restrict the meaning to include only the caudate putamen (CPu), the external globus pallidus (GPe), and the substantia nigra pars reticulata (SNr) [1]. The involvement of CPu, GPe, and SNr in movement disorders in neurological diseases, such as Parkinson's disease and Huntington's disease was widely investigated [2–6]. Previous studies have proposed that basal ganglia circuits are involved in the regulation of purposeful voluntary movement and various functions [7, 8], and dysfunction of basal ganglia leads to movement disorders [9]. Thus, it is important to find some methods of animal models to investigate the pathophysiology of these diseases and screen potential treatments. Although many species have been used to develop experimental models, such as zebrafish [10, 11], rat [12, 13], and primates [14], mouse has become the focus of studies on the function of genes. Up to date, the effects of basal ganglion on movement function and the specific molecular mechanisms remain elusive. The transient receptor potential (TRP) channel superfamily is composed of a set of non-selective cation channels that are responsible to respond to the changes in the external environments. TRP channels compose of several groups that include the TRPC, TRPV, TRPM, etc [15, 16]. TRPC1 was highly expressed in endoplasmic reticulum (ER) [17]. ER Ca^2+^ ion is critical for the regulation of protein translation and some other molecular processes [18]. ER Ca^2+^ dishomeostasis, including ER Ca^2+^ depletion or inhibition of N-linked glycosylation, causes abnormal accumulation of unfolded proteins, thus triggering ER stress [19]. Moreover, changes in Ca^2+^ in ER or mitochondria were able to influence neuronal survival [20]. ER Ca^2+^ depletion activates the store-operated Ca^2+^ (SOC) entry and thus triggers Ca^2+^ entry from the external media *via* the store-operated Ca^2+^ entry (SOCE) [21, 22]. SOC-mediated Ca^2+^ entry, a critical process to maintain ER Ca^2+^ levels, is significantly correlated with TRPC1. Although the molecular identity of the SOCE channel is not yet determined, TRPC1 has been demonstrated to be activated by store depletion *per se* [23, 24]. Moreover, TRPC1 is important for some critical biological and pathological processes such as cell proliferation, axonal guidance, and apoptosis. TRPC1^−/−^ mice has widespread loss of DA neurons and brain tissues of PD patients also show a decreased TRPC1 levels [25]. Besides, TRPC1 is also widely expressed in many regions of the brain such as cerebral cortex, hippocampus, cerebellum, amygdale and in the substantia nigra [26, 27], indicaitng that TRPC1 could have significance in the survival and function of neuronal cells.

In this work, we studied the influence of TRPC1 depletion on motor function and neuronal survival in mice. These data revealed that movement deficits and widespread neuronal loss including dopaminergic (DA) neurons in basal ganglia in TRPC1^−/−^ mice.

## RESULTS

### Movement disorder was caused by TRPC1 depletion

**Figure 1:**
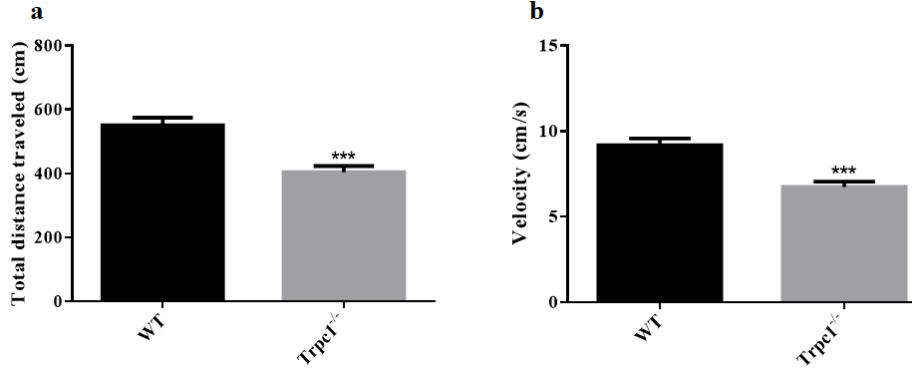
The Movement ability in deep water was measured by swimming test **a.** The total distance traveled; **b.** The average velocity. The data were presented as mean ± SEM. ^***^P < 0.001, *vs*. WT. *n* = 9 for each group.

In order to determine the effect of TRPC1 depletion on motor ability of mice in deep water, swimming test was performed. We measured the total distance traveled and the average velocity of the mice. TRPC1^−/−^ mice showed reduced total traveled distance (*P* < 0.001) (Figure 1a) and decreased average velocity (*P* < 0.001) (Figure 1b) relative to the WT mice. These data suggested that TRPC1 depletion impaired the motor ability of mice in deep water.

**Figure 2:**
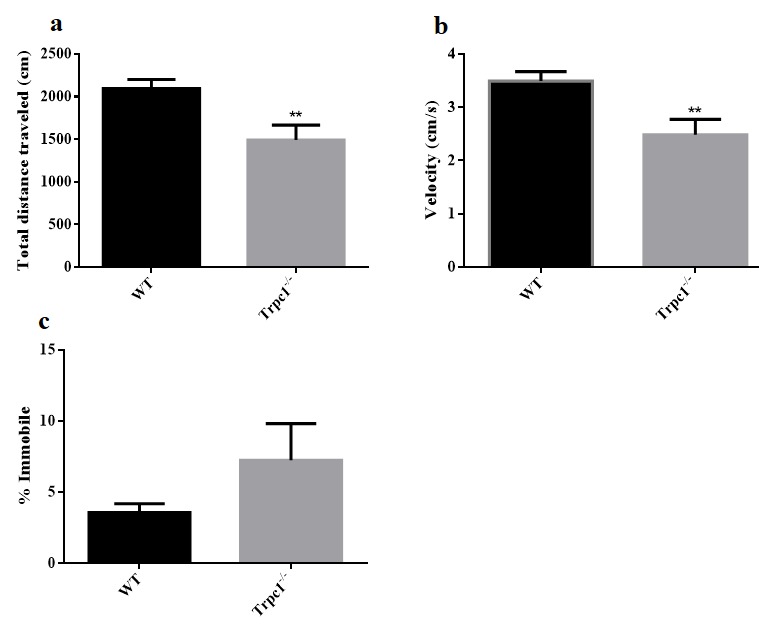
Spontaneous locomotor activity was measured by modified open field **a.** The total distance traveled; **b.** The average velocity; **c.** The time of immobility. The data were presented as mean ± SEM. ^**^P < 0.01, *vs*. WT. *n* = 9 for each group.

In order to determine the effect of TRPC1 depletion on spontaneous locomotor activity of the mice, modified open field test was performed. We found significant differences in the total traveled distance (Figure 2a) and the average movement speed (Figure 2b) between the two groups. The traveled distance and average velocity were significantly decreased in TRPC1^−/−^ mice compared to the WT mice (*P* < 0.01). However, no significant difference in the time of the immobility of the mice was observed in TRPC1^−/−^ mice compared to the WT mice (Figure 2c). These data suggested that TRPC1 depletion impaired the spontaneous locomotor activity of the mice.

**Figure 3:**
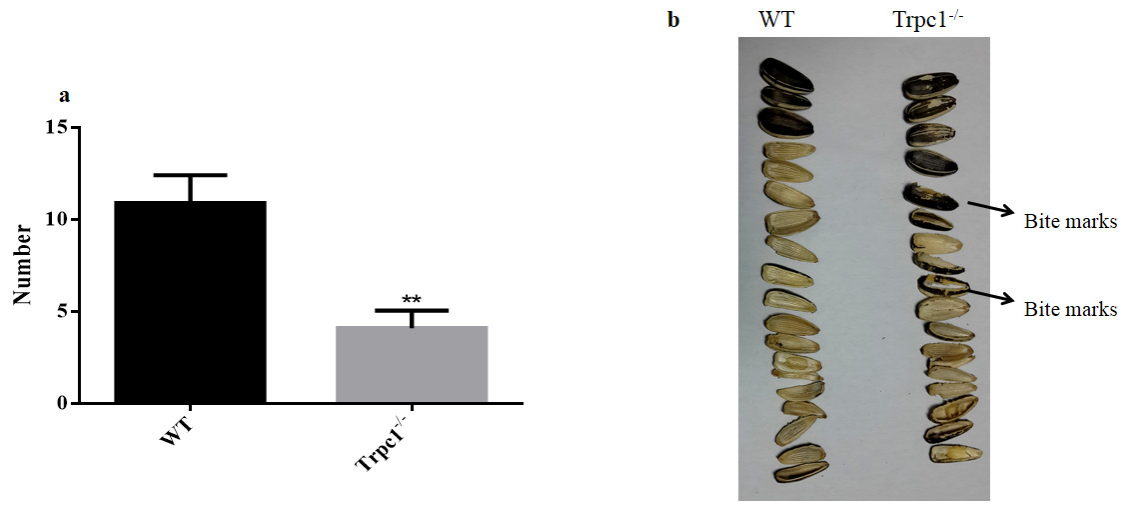
The skilled movement was measured by sunflower seeds eating test **a.** Changes the number of sunflower seeds eaten by the mice during 24 h. **b.** The morphological of sunflower seeds eaten by the mice during 24 h. The data were presented as mean ± SEM. ^**^P < 0.01, *vs*. WT. *n* = 10 for each group.

In order to determine the effect of TRPC1 depletion on the skilled movement of the mice, sunflower seeds eating test was performed. As shown in Figure 3, TRPC1^−/−^ mice ate significantly fewer sunflower seeds during the observation period compared to the WT mice (*P* < 0.001). Furthermore, the eaten sunflower seeds by TRPC1^−/−^ mice displayed significant bite marks. These data indicated that TRPC1 impaired the skilled movement ability of the mice.

### The effect of TRPC1 depletion on neuronal survival in basal ganglia

**Figure 4:**
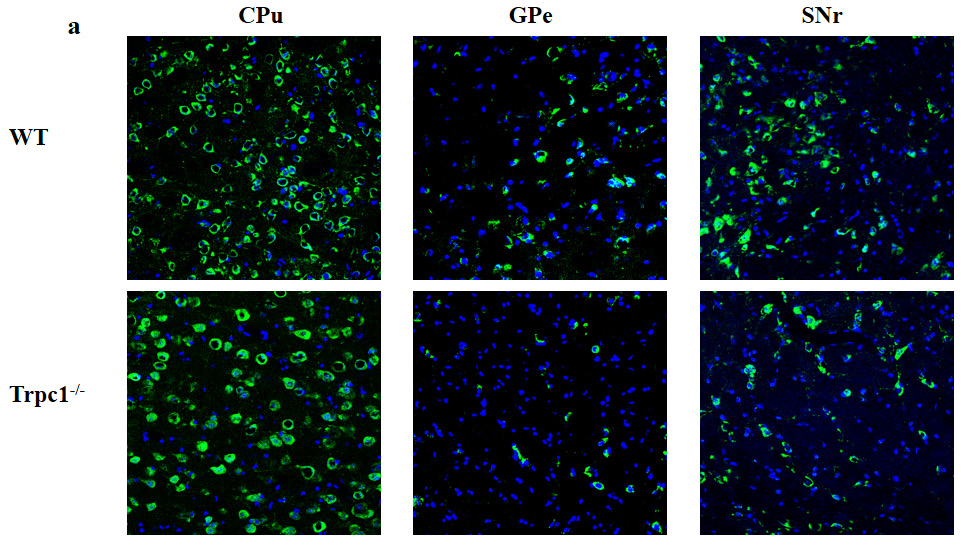

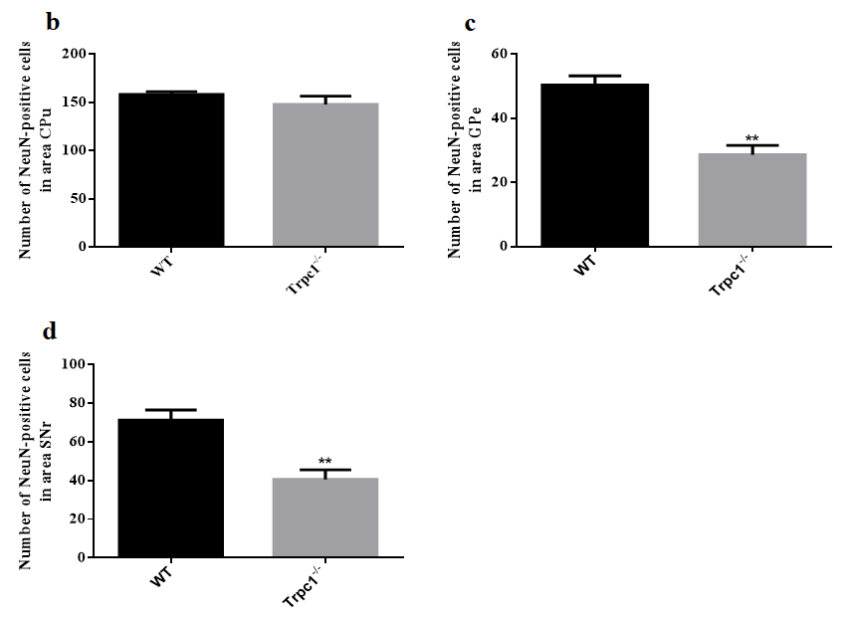
RPC1 depletion caused neuronal loss in basal ganglia T**a.** Immunofluorescent images representing the number of neurons as evidenced by NeuN-staining; **b.** The number of NeuN-positive cells in CPu; **c.** The number of NeuN-positive cells in GPe; **d.** The number of NeuN-positive cells in SNr. The data were presented as mean ± SEM. ^**^P < 0.01, *vs*. WT. *n* = 3 for each group. Scale bar = 100 μm.

In order to determine the effect of TRPC1 depletion on neuronal survival in basal ganglia, i.e. the areas CPu, GPe, and SNr. Immunofluorescence staining was performed using neuron-specific marker NeuN. The data showed that the number of NeuN-positive cells was significantly decreased in GPe and SNr of TRPC1^−/−^ mice relative to the WT mice (*P* < 0.01) (Figure 4a, 4c, 4d), while there was no significant difference of the number in area CPu between the groups (Figure 4a, 4b). These data suggested that TRPC1 depletion caused neuronal loss to some extent in basal ganglia, indicating that TRPC1 is required for the survival of neurons in basal ganglia.

### The effect of TRPC1 depletion on the survival of DA neurons in basal ganglia

**Figure 5:**
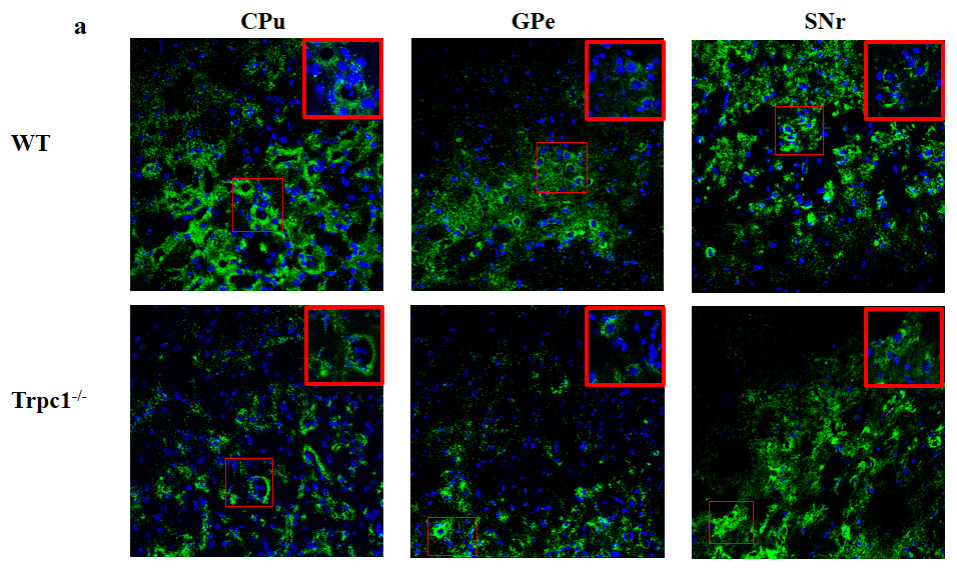

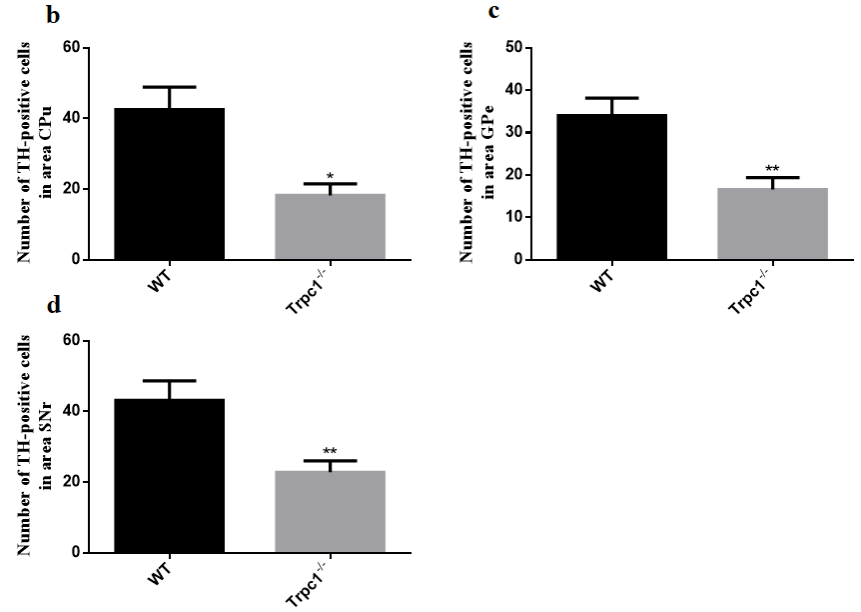
TRPC1 depletion caused the loss of DA neurons in basal ganglia **a.** Immunofluorescent images representing the number of TH-positive cells as evidenced by TH-staining; **b.** The number of TH-positive cells in CPu; **c.** The number of TH-positive cells in GPe; **d.** The number of TH-positive cells in SNr. ^*^ and ^**^P < 0.05 and *P* < 0.01, *vs* WT, respectively. The data were presented as mean ± SEM. *n* = 3 for each group. Scale bar = 100 μm.

In order to determine the effect of TRPC1 depletion on the survival of DA, we performed immunofluorescence using tyrosine tydroxylase (TH) antibody, a maker of DA neurons. We found that TRPC1^−/−^ mice showed significant loss of TH-positive cells relative to the WT mice in CPu (*P* < 0.05) (Figure 5a, 5b), and the number of TH-positive cells was also significantly decreased in GPe and SNr (*P* < 0.01) (Figure 5a, 5c, 5d). These data suggested that TRPC1 depletion caused loss of DA neurons in basal ganglia.

### TRPC1 deficiency caused neuronal apoptosis in basal ganglia

**Figure 6:**
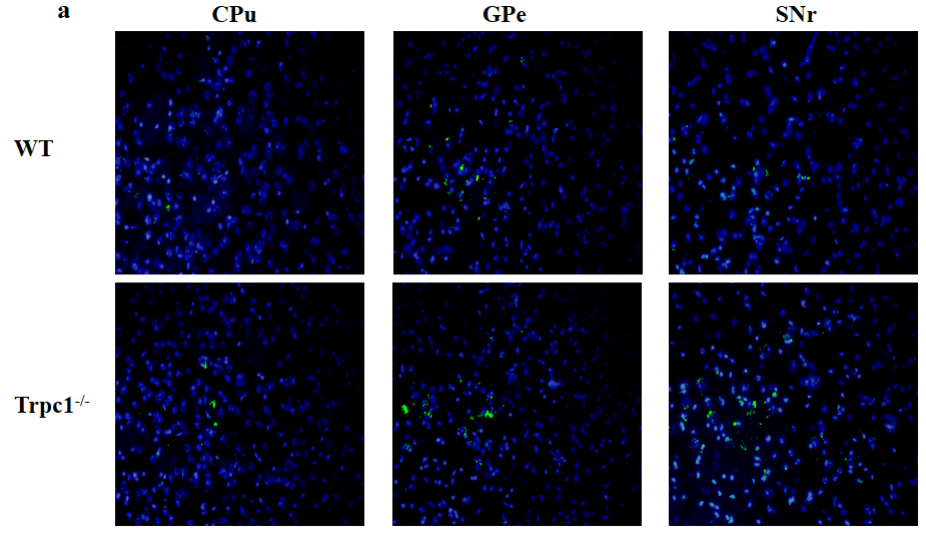

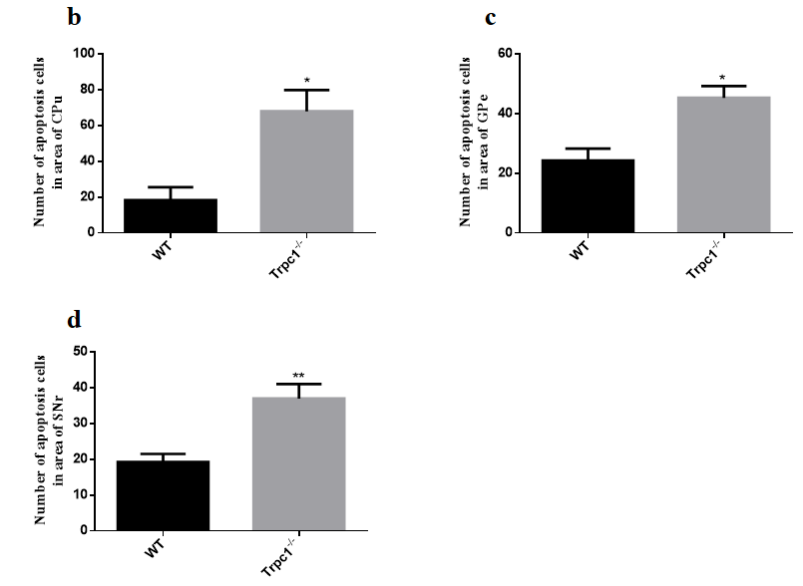
TRPC1 depletion caused neuronal apoptosis in basal ganglia **a.** A representative image of neuronal apoptosis in basal ganglia; **b.** The number of apoptotic cells in CPu; **c.** The number of apoptotic cells in GPe; **d.** The number of apoptotic cells in SNr. ^*^ and ^**^P < 0.05 and *P* < 0.01, *vs* WT, respectively. The data were presented as mean ± SEM. *n* = 3 for each group. Scale bar = 100 μm.

In order to determine the effect of TRPC1 depletion on apoptosis, we performed TUNEL staining to measure the change of apoptosis in basal ganglia. The data showed significantly increased TUNEL-positive cells in CPu (*P* < 0.05), GPe and SNr (*P* < 0.01) of TRPC1^−/−^ mice compared with the WT mice (Figure 6a-6d), suggesting that neuronal loss caused by TRPC1 depletion may be a consequence of neuronal apoptosis in basal ganglia.

## DISCUSSION

Previous studies have shown that TRPC1 is vital for neuronal survival [27, 28], indicating that TRPC1 may be involved in the regulation of motor activity. In this study, we demonstrated that depletion of TRPC1 caused movement disorder of mice and the loss of NeuN neurons and DA neurons in basal ganglia, including the area of CPu, GPe and SNr.

The behavioral tests, i.e. swimming test, modified open field test, and sunflower seeds eating test, consistently demonstrated that TRPC1 depletion caused movement disorder in mice. Motor function in swimming activity requires a different set of muscles, spinal reflexes, and brain regions, which is different from the movement function involved in locomotion, wheel running and some other motor behaviors [29]. Additionally, there are two major disadvantages for swimming test of movement function: (i) the animals may drown in highly immobile state; (ii) swimming in cold water may trigger stress, and may affect the outcome [30]. In this study, we maintained the water temperature at 24 ± 2°C to overcome this problem. Simultaneously, TRPC1^−/−^ mice showed DA neuronal loss in the SNr, a brain region closely linked with PD [25].

Spontaneous activity of animals was measured by modified open field test which had been used to assess basal ganglia related movement disorder in mice [31]. The advantages of this method were as follows: (i) weak light environment was more aligned with living habit of the mice, then more conducive to free moving, (ii) the 20 minutes travelling increased the difficulty of movement, thus making the differences in motor function more obvious. Sunflower seeds eating was used to assess skilled movement function. Normal mice executed skilled movement with seemingly effortless precision, depending on elaborate series of neural transformations by the interaction of different neurons especially DA neuron [32]. Our present study showed that TRPC1 depletion impaired the movement function of the mice, especially skilled movement function. Besides, previous studies also demonstrated that TRPC1 depletion could cause spatial memory impariment of mice [33].

NeuN is widely used as a neuronal marker [34]. NeuN was reported to be significantly reduced in SOD1 mice with movement deficit [35]. Additionally, BDNF could promote survival of basal ganglia neurons and its progressive loss had been shown in neurodegenerative diseases such as HD [35]. Thus to some extent, the decrease of NeuN neurons could reflect down-regulation of BDNF. In our study, NeuN neurons were significantly decreased in GPe and SNr areas of TRPC1^−/−^ mice compared to the WT mice. Taken together, NeuN neurons may be necessary for motor functions.

Another important finding in the present study was that histopathological analysis demonstrated significant loss of DA neurons in basal ganglia of TRPC1^−/−^ mice, including the CPu, GPe, and SNr areas. In c-rel^−/−^ mice, researchers found that motor deficits was observed responsive to L-DOPA treatment, which was associated with DA neuronal loss in the SNr [36].Besides, injection of 6-OHDA into the CPu area caused motor impairment, and rats with combined DA neurons lesions were more prone to suffering dyskinesia [37]. These data indicated that DA neurons in basal ganglia especially the GPe and SNr area played a vital role in the regulation of motor function. The GPe area was critical for the regulation of motor function, and a previous study also showed that DA neuron-depletion in the GPe area induced significant movement impairment [38]. In our study, TRPC1 depletion was shown to cause movement disorder in the mice, which could be a consequence of the loss of the DA neurons in basal ganglia caused by TRPC1 depletion.

To further determine whether the neuronal loss was related to apoptosis, we evaluated the effects of TRPC1 depletion on neuronal apoptosis using TUNEL staining. The results showed that TRPC1 depletion could increase the number of TUNEL-positive neuronal cells in basal ganglia, suggesting that neuronal loss caused by TRPC1 depletion as observed may result from neuronal apoptosis. Besides, a study reported that TRPC1 could prevent human SH-SY5Y cells from cytotoxicity by inhibiting cellular apoptosis [39]. In some neurodegenerative diseases such as AD and PD, apoptosis is the main cause leading to neuronal death [40], and preventing apoptotic processes is a strategy for the treatment of these diseases [41, 42]. Thus, it was indicated that the loss of DA neurons, resulting from cell apoptosis, consequently caused movement disorder by TRPC1 depletion.

In summary, this study demonstrated that TRPC1 depletion caused movement disorder in mice and neuronal loss including DA neurons in basal ganglia. TUNEL staining indicated that neuronal apoptosis may contribute to neuronal loss. Our data suggested that TRPC1 was involved in the control of motor function by regulating the survival of neuronal cells of basal ganglia.

## MATERIALS AND METHODS

### Animals

The TRPC1^−/−^ mice were obtained from Prof. Lutz Birnbaumer (NIEHS, US) and the wild type (WT) mice were purchased from Vital River Laboratory Animal Technology Co. Ltd (Beijing, China). A total of thirty mice at 5 months old were used in this study. All the mice were randomly divided into WT mice and TRPC1^−/−^ mice. The mice were housed in groups of 15 mice per cage (470×350×200mm), and kept under controlled light and environmental conditions (12-hour light / 12-hour dark cycle with light on from 6: 00 am to 18: 00 am at a constant temperature of 24 ± 2 °C and humidity) with free access to food and water. Animal treatment and care procedures were performed following the National Institutes of Health Guide for the Care and Use of Laboratory Animals (NIH publications no. 80-23) and the Regulations for Animal Care and Use Committee of Experimental Animal Center at Shenzhen Center for Disease Control and Prevention. All efforts were made to minimize animal suffering and reduced the number of mice used.

### Swimming test

The mice were subjected to the swimming test as previously described [29]. The behavioral test was carried out in a circular pool (height 30 cm, diameter 120 cm) filled with water (temperature of 24 ± 2 °C) to a level that prevented the mice from touching the bottom. The mouse was placed in a fixed position in the pool, facing the tank wall, then was released into the water at the water-level (not dropped). After 1 min, the mouse was wiped dry immediately using a dry cloth and returned to a cage to have 1-min break. The test was repeated 4 times. The pathway that the mice went through was recorded by a video camera that was connected with a digital-tracking instrument attached to the computer loaded with the software (Huaibei Zhenghua Biologic Apparatus Facilities Limited Company, Huaibei, Anhui, China).

### Modified open field test

The experiment was performed in a sound proof, weak light environment as previously described [43]. The apparatus consisted of a rectangular area of 50×50 cm, with plastic walls of 40 cm height and white floor that was divided equally into 16 compartments. The animals were placed individually in one corner of the open field, facing the center. Each mouse was in the area for a total of 20 min, with 10 min for acclimation, and 10 min for data acquisition. The total distance traveled, the average velocity, and the time of immobility were calculated. After each test, the apparatus was cleaned with 75% (v/v) alcohol to avoid the residual odor.

### Sunflower seeds eating test

Sunflower seeds eating test was used to assess skilled movement ability. Before the formal experiment, each mouse was placed into a single cage in order to adapt to the new environment. Then sunflower seeds eaten by the animals were analyzed. On the first day, 20 sunflower seeds were put into each cage. After 24 h, the number of seeds was recorded and the morphological of the sunflower seeds were analyzed.

### Sample preparation for tissue sections

After behavioral tests, the mice were anesthetized with 4% chloral hydrate and perfused *via* the left cardiac ventricle with 0.9% NaCl, followed by 4% (w/v) paraformaldehyde fixative in 0.1 M phosphate buffer solution (pH = 7.4), then the brains were removed and stored in 4% (w/v) paraformaldehyde fixative at 4 °C for 18 h. The whole brains were placed in a 30% sucrose solution in 0.1 M phosphate buffer solution for further use.

After fully fixation, the tissues were embedded in O. C. T. compound (Leica Cat. No. 14020108926), and subjected to cryosectioning at a thickness of 15 μm. Continuous cryosectioned slices were placed on 0.1 M phosphate buffer solution (pH = 7.4), and were selectively mounted on adhesion microscope slides (CITOGLAS. Ref 188105W) according to different experimental requirements (CPu or GPe or SNr), to minimize sample variations. The collected sections were stored at −20 °C until further use.

### Immunofluorescent staining

Immunofluorescence staining was performed as previously described [44]. In brief, 15 μm-thick coronal sections were rinsed in PBS 4 times for 5 min. The sections were blocked for 60 min in blocking buffer solution (1×PBS / 5% normal serum / 0.3% Triton ^TM^ X-100). Primary antibodies used were against Anti-NeuN (rabbit monoclonal, diluted 1:500) and tyrosine hydroxylase (TH) (rabbit polyclonal, diluted 1:200) (both Cell Signaling Technology, USA). Incubation with primary antibodies were performed overnight at 4 °C in primary antibody dilution buffer (1×PBST with 5% BSA). After being washed with PBST, the sections were stained with the secondary antibody, Alexa Fluor^®^-488 goat anti-rabbit (Invitrogen) (1:500). The sections were incubated for 1 h in the dark at room temperature, then stained with DAPI (Beyotime Institute of Biotechnoloy, Haimen, China) for 1 min to reveal the nuclei. The brain sections were examined under a laser scanning confocal microscope (Leica, Wetzlar, Germany).

### TUNEL staining

For the detection of apoptosis, TUNEL staining was performed to determine neuronal apoptosis using the DeadEnd^TM^ Fluorometric TUNEL System (Promega). Briefly, the brain sections were immersed in 0.85% NaCl for 5 min and washed with 1×PBS for 5 min at room temperature, then the sections were fixed by immersing in 4% paraformaldehyde fixative for 15 min and washed with 1×PBS for 5 min. After the excess liquid was removed, the sections were treated with 100 μl of the 20 μg/ml proteinase K to each slide and incubated for 10 min at room temperature. While the sections were equilibrating, the Nucleotide Mix was on ice and sufficient rTdT incubation buffer were prepared for all experimental reactions, then 50 μl rTdT incubation buffer was added to each slide. The sections were covered with plastic coverslips to ensure even distribution of the reagent. The DNA fragments were labeled with the rTdT at 37 °C for 1 h in the dark. The sections were stained with DAPI for 3 min and observed by a microscope (Olympus 1X51, Tokyo, Japan).

### Statistical analysis

Statistical analysis was performed using GraphPad Prism 6.0 statistical software (GraphPad Software, Inc.). The significance of the differences between the groups was determined by unpaired *t*-test. The data were expressed as mean ± standard error (SEM). The level of significance was set at *P* < 0.05.
